# Depressive symptoms in the general population before and in the first year of the COVID-19 pandemic: Results of the GEDA 2019/2020 study

**DOI:** 10.25646/10664

**Published:** 2022-12-20

**Authors:** Ulfert Hapke, Christina Kersjes, Jens Hoebel, Ronny Kuhnert, Sophie Eicher, Stefan Damerow

**Affiliations:** Robert Koch Institute, Berlin Department of Epidemiology and Health Monitoring

**Keywords:** COVID-19 PANDEMIC, DEPRESSION, MENTAL HEALTH, RESILIENCE, AGE, PHQ-8

## Abstract

**Background:**

Study results on the impact of the COVID-19 pandemic on mental health in the first year of the pandemic are contradictory. The GEDA 2019/2020 study makes it possible to examine changes in depressive symptoms in the population.

**Methods:**

A standardised telephone interview was used to survey a random sample of the population in Germany aged 15 and older. To exclude seasonal effects, 10,220 interviewees from the period April 2019 to January 2020 were compared with 11,900 from the period April 2020 to January 2021. Depressive symptoms were assessed with the internationally established 8-item Patient Health Questionnaire (PHQ-8).

**Results:**

The prevalence of depressive symptoms decreased from 9.2% to 7.6% in the first year of the pandemic. Changes differ between women and men as well as between age and education groups. The analysis of individual symptoms suggests that it is not about a reduction of mental disorders of the depressive type in the narrower sense, but rather a decrease in stress-associated individual symptoms.

**Conclusions:**

The decrease in stress-associated depressive symptoms in parts of the population can be interpreted as an indication that pandemic-related changes in everyday life and the working environment may have had a positive effect on individual areas of mental health in certain groups, at least temporarily in the first year of the pandemic. The continuing strong social inequality in depressive symptoms to the disadvantage of low education groups confirms that the need for social situation-related health promotion and prevention with regard to the living and working conditions of socially disadvantaged people must not be lost sight of in times of pandemic. For groups in the population that partly showed a worsening of symptoms in this phase of the pandemic, e.g. the diminished ability to concentrate of very old men, targeted support options should be created in the future.

## 1. Introduction

As a population-representative health survey among adults in Germany, the study German Health Update (GEDA) forms an essential pillar of the continuous health monitoring at the Robert Koch Institute (RKI) [1]. Since GEDA 2014/2015-EHIS, the questionnaire of the European Health Interview Survey (EHIS), which is conducted every five years to take stock of the health situation in the population aged 15 and older, has been integrated into GEDA [2]. The current work complements previous work on depressive symptoms, which was based on GEDA-EHIS. Among other things, depressive symptoms were described together with other selected indicators of the health situation in Germany [3], results for Germany were compared with other European countries [4] and first observations since the beginning of the COVID-19 pandemic were published [5–7].

Depressive symptoms do not only occur in the case of manifest depression in the sense of a mental disorder. It can also occur as an accompanying secondary symptom of other mental disorders, trauma, chronic stress and other psychological distress. The consequences of depression for the individual, society and the health system are serious [8]. For this reason, it is of particular importance to identify any changes in the population triggered by crises. The questionnaire used in GEDA 2019/2020-EHIS, the Patient Health Questionnaire (PHQ-8), is correlated with almost all areas of mental health and covers a total of eight symptom areas. This instrument makes it possible to analyse specific symptom areas and to attribute any changes in depressive symptoms to individual symptom areas. Basic results on depressive symptoms in connection with other aspects of health, methodological features of the PHQ and its significance in comparison to other population-related indicators of mental health in general as well as depression in particular, have been published in a current focus report on mental health in Germany [8]. The current GEDA survey allows an analysis of depressive symptoms during, as well as a comparison with times before the pandemic.

Prior to the present analyses, the RKI prepared a rapid review (as of July 30, 2021) on the mental health of adults in Germany during the COVID-19 pandemic [9]. At that time, the majority of the studies referred to the first phase of the pandemic, which was mainly characterised by containment measures and the associated changes in care. For the most part, the studies showed both an overall resilient population and largely adaptive care [10]. However, there were indications of vulnerable subgroups. It is important to note that the review and subsequent published works also examined studies of other indicators of mental health, such as subjectively experienced stress, loneliness, quality of life and anxiety symptoms. Although these findings are not readily transferable to depressive symptoms, due to overlaps and additions, they are very helpful in analysing depressive symptoms in the context of the pandemic.

In summary, the following observations were made: Women tended to show abnormalities in loneliness [11–13], anxiety [14, 15], depressive symptoms [13], depressive and anxiety symptoms [11], and lower affective well-being [11, 13] and life satisfaction [11]. Women rate their resilience as lower [16]. Professional absenteeism due to mental disorders also increased in 2020, especially among women, but this is embedded in a general trend of the years before [17]. People under the age of 30 appear to be affected more often or more severely by the effects of the pandemic, according to previous publications. They are more affected by loneliness [11, 12, 18], depressive symptoms [19] and depressive and anxiety symptoms [11]. They also rate themselves as less resilient compared to the norm values available for Germany [20, 21]. With regard to anxiety symptoms, age differences seem to be less pronounced at the beginning of the pandemic [15], while younger people are more affected in later phases [19, 22]. These short-term consequences have been interpreted in younger people more as reactions to stress and less as mental disorders in the narrower sense [16]. A scoping review on the situation of older people in the initial phase of the COVID-19 pandemic gives indications that elderly were particularly affected by the contact restrictions associated with the pandemic [23]. For Germany, however, there is hardly any reliable data, especially for people living in private households.

The findings on mental health over the course of the pandemic are consistent in many respects for both sex and age. However, there is still a lack of methodologically high-quality data from representative samples for later pandemic phases as well as meaningful longitudinal and trend studies that map a course and include the time before the pandemic as a comparison.

Against the background of the studies and data available so far, there is little information on whether and how the frequency of depressive symptoms in women and men and in different age groups has changed. Also, little is known about whether education proves to be a resource of resilience in the pandemic.

The results so far mainly refer to the first phase of the pandemic. However, a further analysis beyond the period of the beginning of the pandemic seems advisable against the background of a dynamic change of the pandemic situation and the possible consequences. Since initial analyses of the prevalences of depressive symptoms show fluctuations in the course of the year [6, 7], the present analysis is intended to compare two calendar periods of time before and during the pandemic that are largely identical.

In previous studies in which the PHQ-8 or PHQ-9 was used, the overall test result on depressive symptoms was reported. In this study, however, the individual symptom areas are examined. This is intended to determine whether any effects on depressive symptoms can be attributed to individual symptom areas. The analyses are stratified by gender and different age and education groups in order to find out whether differences between parts of the population can be identified.

**GEDA 2019/2020**

Fifth follow-up survey of the German Health Update

**Data holder:** Robert Koch Institute

**Objectives:** Provision of reliable information on the health status, health behaviour and health care of the population living in Germany, with the possibility of European comparisons

**Study design**: Cross-sectional telephone survey

**Population:** German-speaking population aged 15 and older living in private households that can be reached via landline or mobile phone

**Sampling:** Random sample of landline and mobile telephone numbers (dual-frame method) from the ADM sampling system (Arbeitskreis Deutscher Markt- und Sozialforschungsinstitute e.V.)

**Sample size:** 26,507 respondents

**Study period:** April 2019 to January 2021 (GEDA-EHIS to September 2020)

**GEDA survey waves:**

► GEDA 2009
► GEDA 2010
► GEDA 2012
► GEDA 2014/2015-EHIS
► GEDA 2019/2020

Further information in German is available at www.geda-studie.de

## 2. Methods

### 2.1 Study design and sampling

The GEDA study is a cross-sectional survey based on a nationwide random sample of the adult resident population living in Germany. Commissioned by the Federal Ministry of Health, the GEDA study has been conducted by the RKI at multi-year intervals since 2008 and is part of the health monitoring system [24, 25]. The current GEDA survey is a telephone survey of the German-speaking population aged 15 years and older using a programmed, fully structured questionnaire (Computer Assisted Telephone Interview) (Info box). Details on the range of topics, questionnaire and sample design, sampling and data weighting of the GEDA 2019/2020-EHIS study are described in detail elsewhere [26, 27].

After the originally planned survey was completed in September 2020, it was possible to continue the data collection from October 2020 until January 2021 in order to observe the effects of the pandemic as it progressed. The study design was maintained with a slightly shortened questionnaire. The survey period from April 2019 to January 2021 is referred to as GEDA 2019/2020, whereas GEDA 2019/2020-EHIS refers to the detailed questionnaire up to the study date of September 2020.

A total of 23,001 people participated in the GEDA 2019/2020-EHIS study with complete interviews. The continuation of interviews between October 2020 and January 2021 includes 3,506 participants. A total of 26,507 people (13,955 female, 12,552 male) participated in GEDA 2019/2020 between April 2019 and January 2021.

### 2.2 Indicator and analysis groups

#### Depressive symptoms

The presence of depressive symptoms in the last two weeks was assessed by self-reporting by the participants using the internationally established 8-item Patient Health Questionnaire (PHQ-8) [28].

This instrument assesses eight symptom areas of major depression in line with the Diagnostic and Statistical Manual of Mental Disorders (DSM-IV, 4. Edition) [29]. The initial question is: ‘Over the last 2 weeks, how often have you been bothered by any of the following problems?’ The eight symptom areas are as follows: 1. Little interest or pleasure in doing things; 2. Feeling down, depressed, or hopeless; 3. Trouble falling or staying asleep, or sleeping too much; 4. Feeling tired or having little energy; 5. Poor appetite or overeating; 6. Feeling bad about yourself, or that you are a failure, or have let yourself or your family down; 7. Trouble concentrating on things, such as reading the newspaper or watching television; 8. Moving or speaking so slowly that other people could have noticed. Or the opposite – being so fidgety or restless that you have been moving around a lot more than usual. For each item, the frequency is asked with the categories ‘not at all’, ‘several days’, ‘on more than half the days’, or ‘nearly every day’. The frequencies are given the value 0 to 3 and the presence of depressive symptomatology is assumed from a scale total value of at least 10 of the maximum 24 points. When evaluating the individual questions, dichotomisation was used: ‘not at all’ or ‘several days’ was analysed as inconspicuous and ‘on more than half the days’ or ‘nearly every day’ was analysed as impaired.

#### Education

Education status was determined by highest school-leaving qualification and highest professional qualification of respondents. The CASMIN classification (Comparative Analysis of Social Mobility in Industrial Nations) was used to distinguish between a low (CASMIN 1: primary or low secondary education), medium (CASMIN 2: medium or high secondary education) and high (CASMIN 3: tertiary education) education group [30].

#### Gender

The analyses for women and men were based on the information provided by the respondents and not on biological sex [31].

#### Age groups

When forming the age groups, a rough subdivision was chosen in favour of statistical significance.

### 2.3 Statistical analysis

In a first step, the sample was divided into two comparison periods, the period before (April 2019 to January 2020) and from the beginning of the pandemic (April 2020 to January 2021). Only identical interview weeks were used, which are available at both periods, in order to exclude any seasonal effects on the indicators. Since data were only collected in the first few days of January in both 2020 and 2021, the year jump in the designation of the periods is omitted in the text for better readability. These were participants who were interviewed in the first week of January due to the December holidays. In addition, all equivalent interview weeks in September and October 2019 are excluded, as data collection in 2020 was interrupted for six weeks in these months.

The analyses are thus based on data from 22,120 participants aged 15 to 101 years. Among the respondents were 11,670 women, 10,386 men and 64 respondents who indicated a different or no gender. In the analyses by gender, the latter are not shown separately because the case numbers are too small. However, they are included in the overall category [31].

To correct for deviations of the sample from the population structure, the analyses were performed applying a weighting factor. As part of the data weighting, a design weighting was first performed for the different selection probabilities (mobile and landline network). This was followed by an adjustment to the official population figures based on age, sex, federal state, and district type (as of: 31/12/2019). In addition, the sample was adjusted to the education distribution in the 2017 Microcensus according to the ISCED classification [32]. The weighting procedure is described in detail elsewhere [26].

In addition, the probability of participation of certain population groups could be influenced due to the COVID-19 pandemic and the associated containment measures (e.g. home office, contact restrictions) [7, 33]. For this reason, as described in [7] an additional adjustment weighting was performed separately for the sample periods before and after the cut-off date of 16.03.2020 (adoption of the federal states agreement on guidelines against the spread of the corona virus [34]). For the analyses stratified according to education groups, age standardisation to the European Standard Population 2013 was performed, in order to compensate for cohort effects with regard to educational qualifications and corresponding age differences between the education groups.

The analyses were performed with SAS 9.4. All analyses were calculated using the SAS survey procedures of in order to take the appropriate weighting into account when calculating confidence intervals and p-values. A statistically significant difference between groups/time periods is assumed if the corresponding p-value in the Rao-Scott-Chi-Square test is smaller than 0.05.

## 3. Results

### 3.1 Depressive symptoms

Depressive symptoms according to PHQ-8 were present in 9.2% of the respondents before the pandemic (Table 1). During the pandemic, the prevalence in the total sample was lower at 7.6%. However, the prevalence decreased statistically significantly only among women from 9.8% to 7.6%. In men, the prevalence before the pandemic was 8.5% and was comparable to that in women at 7.4% during the pandemic. The strongest declines occurred among women in the age groups 30 to 44 and 45 to 64 years. Overall, there is a parallel trend in the age groups up to 64 years of age. This trend is not confirmed in the age groups from age 65 and older.

The analysis differentiated by education shows considerable education differences in depressive symptoms in both periods, with the highest prevalences in the low education groups (Table 2). Overall, these strongly pronounced differences to the disadvantage of low education groups persist over the observation period from before to during the pandemic. In the low and medium education groups, there is a declining trend in depressive symptoms, however this is only significant in the largest group with medium education status when women and men are analysed together. The group with high education has a consistently low prevalence of depressive symptoms both before and during the pandemic compared to the medium and low education groups.

### 3.2 Individual symptom areas of depressive symptoms

The analyses of the individual symptoms of the PHQ-8 are contained in Table 3. With regard to the two core characteristics of depressive symptoms 1. ‘Little interest or pleasure in doing things’ and 2. ‘Feeling down, depressed, or hopeless’, the analyses do not reveal a clear trend in the overall sample when comparing the two time periods. However, when subdivided by sex and age groups, a partially opposing development is shown. The increase in the frequency of question 1 among respondents aged 80 and older is striking. Among women, the percentage increases from 8.8% to 12.4% and among men from 9.1% to 16.0%. Due to the small number of cases of people aged 80 and older, an additional test was performed here to see to what extent an age effect could be observed without differentiating between women and men. This resulted in an increase from 8.9% to 13.9% (p=0.029), which deviates from the results in younger age groups, with the exception of women aged 15 to 29 years.

During the pandemic, compared to the year before the pandemic, there is a significant decrease in frequency for symptoms 4. ‘Feeling tired or having little energy energy’, 5. ‘Poor appetite or overeating’ and 6. ‘Feeling bad about yourself – or that you are a failure or have let yourself or your family down’.

For women, the figure was at least on half of the days for 4. Suffering from ‘Feeling tired or having little energy’ decreased from 20.3% to 15.2%. The greatest decrease from 26.2% before the pandemic to 17.0% during the pandemic was among women aged 15–29. Only for women aged 80 years and older was no decline reported. There were no significant changes among men, but the prevalences among 15 to 29 year olds, 65 year olds and older with an increase in symptoms trended in the opposite direction to the middle age groups of 30 to 64 years.

The frequency of 5. ‘Poor appetite or overeating’ showed a decrease from 13.3% to 7.4% among women in the age group of 15 to 29 years. Due to the opposite trend among women in the age groups 65 and older compared to the younger age groups, a supplementary analysis was performed summarising these age groups. This showed an increase in the time of the pandemic from 3.8% to 6.1% (p=0.035). Men showed a decrease from 6.8% to 5.2%, which, unlike for women, was similar in all age groups. ‘Feeling bad about yourself – or that you are a failure or have let yourself or your family down’ (question 6) was significantly less frequent in the gender-specific analysis only among women during the pandemic than before the pandemic (3.0% vs. 46%). A similar development was observed among men, but to a lesser extent (3.7% vs. 4.2%). It is noticeable that among women and men, the question 6. ‘Feeling bad about yourself – or that you are a failure or have let yourself or your family down’ was stated much more frequently among younger people than among older people. In addition, an increase in frequency of 7. ‘Trouble concentrating on things’ among men aged 80 years and older from 3% before the pandemic to 9.8% during the pandemic (p=0.018) can be observed.

The analysis results documented in Table 4 on the individual symptoms surveyed with the PHQ-8 show consistent and time-persistent differences between the three education groups. All individual symptoms are reported most frequently in the low education group and least frequently in the high education group. The decrease in the frequency of 4. ‘Feeling tired or having little energy‘ in the low and medium education groups in the first pandemic year compared to the previous year is striking. The separate analysis by gender shows that this decrease is primarily observed in women. Another change during the pandemic period is the near doubling of 7. ‘Trouble concentrating on things, such as reading the newspaper or watching television’ among men in the high education group from 1.4% before the pandemic to 2.7% during the pandemic. However, despite doubling in this education group, the frequency of this symptom area still remains significantly below the frequency in the other education groups.

## 4. Discussion

### 4.1 Depressive symptoms

With the data from the GEDA 2019/2020 study, a lower prevalence of depressive symptoms according to PHQ-8 is observed in the first year of the pandemic (2020) than in the comparison period one year earlier. The sharpest decrease was seen in women in the age groups 30 to 64 years. It is remarkable that the sex difference in depressive symptoms [8, 35] found in earlier studies does not persist under the conditions of the pandemic. Whereas the latter already existed before the beginning of the pandemic only in the age groups from 15 to 44 years. It is worth mentioning in this context that in the age group of 45 to 64 year-olds, women with 11.3% and men with 11.0% showed almost no differences even before the pandemic, and a uniform decline was observed after the start of the pandemic. A prevalence of 8.2% was found in both men and women. This decline in the 30 to 64 age groups can possibly be explained by the fact that the measures taken during the pandemic, such as home office and short-time work, not only promoted protection against infection, but also brought about a stress-reducing deceleration in the working and living environment [9], which could have had a positive effect, at least temporarily, on individual areas of the mental health of certain population groups during the first year of the pandemic. However, further research is needed to explain this finding. In principle, it is also conceivable that measures accompanying the pandemic, which were intended to counteract psychological distress, have promoted a reduction in depressive symptoms [36].

The considerable social differences in the prevalence of depressive symptoms to the disadvantage of low educated groups remain under pandemic conditions. Contrary to assumptions sometimes made and justified in references [9], population groups with low education apparently had no additional increased risk of developing depressive symptoms under conditions of the pandemic, at least in the first year of the pandemic considered here. However, the socioepidemiological pattern of a higher prevalence of depressive symptoms in low education groups has persisted both before and during the pandemic and corresponds to the pattern already found in previous GEDA waves [35].

### 4.2 Individual symptom areas of depressive symptoms

The analysis of individual symptom areas shows that there are partly contrary developments that are not visible in the overall result for the PHQ-8. For example, among those aged 80 and older, there is an increase in the symptom area ‘little interest or pleasure in in doing things’, in women from 8.8% to 12.4% and in men from 9.1% to 16.0%. Furthermore, 9.8% of men of this age reported diminished ability to concentrate in the first year of the pandemic, compared to 3.0% in the pre-pandemic period, which was significantly lower. Although these results are subject to a relatively large statistical uncertainty and must therefore be interpreted with caution, there seems to be evidence that individuals in this age group living in private households were not only particularly affected by isolation during the pandemic, but in the case of men, may have experienced effects on cognitive performance. It is known from a larger population study on cognitive performance that social support, in the sense of a supportive density of contact, is beneficial to maintaining cognitive performance in old age [8]. In the future, special attention should be paid to ensuring supportive contact services for people who become highly isolated in a pandemic situation.

The decrease in frequency of ‘Feeling tired or having little energy’, ‘Poor appetite or overeating’ and ‘Feeling bad about yourself – or that you are a failure or have let yourself or your family down’ are symptom areas associated with chronic stress [37, 38]. The results support the interpretation that the decline in the frequency of depressive symptoms in the early period of the pandemic is explained by a decline in specific everyday stresses rather than by a decline in individuals with a depressive disorder in the narrow sense. In this context, the OECD has introduced the term ‘Mental Ill-Health’, which is to be understood more as impaired mental health and less as a chronic mental illness [39]. It is also consistent that in the first two symptoms queried in the PHQ-8, 1. ‘Little interest or pleasure in doing things’ and 2. ‘Feeling down, depressed, or hopeless’, no very large changes were found. These two core features need to be present in a severely impairing way in order to diagnose major depression, along with other accompanying symptoms [40]. Fullana et al. assume that the elimination of work-related duties during the pandemic provided a chance for temporary recovery in part of the population [36]. A study with a non-probabilistic sample was able to show that people who worked from home during the pandemic experienced less stress, reported more positive and less negative affect, and more life satisfaction than those who did not work from home [41]. The authors of this study, following the theory of resource maintenance, interpret that home office can be seen as a resource gain and, according to self-regulation theory, a way to cope with the pandemic challenges. A rapid review on this topic emphasises that the potential resource gain from home office during the pandemic and the positive effects on mental health that could be achieved with it depended on how good the organisational support was for people who worked from home during the pandemic [42].

Another indication of the potentially positive effects of changes in working conditions are the results on tiredness and the loss of energy. In 2019, in the age groups up to 64 years, between 20.3% and 26.2% of women reported that they were affected ‘on more than half the days’ or ‘nearly every day’. During the pandemic period, the percentage decreased to between 15.2% to 17.0%. For men, the proportions were significantly lower in 2019 and only among the age groups from 30 to 64 years, although not significant, the trend was downward. This is the phase of life characterised by working life.

These observations are consistent with other study results at the beginning of the pandemic, which also reported a decrease in individual symptoms of depressive symptoms. As in the present study, there was a decrease in symptoms of tiredness and loss of energy [5, 6] as well as diminished ability to concentrate [7].

As part of the National Cohort (NAKO Health Study) [43, 44], a special survey was conducted at the beginning of the COVID-19 pandemic between 30 April 2020 and 15 May 2020, in writing and online, using the PHQ-9 as a survey instrument [43]. The prevalence of depressive symptoms was 9.5% during the survey period, 2.4% higher than the average prevalence of 7.1% in 2014–2019. However, an increase can only be seen in the age groups below 60 years, especially among younger women [44]. On the other hand, 32% of the respondents also reported an improved self-assessed health status at the beginning of the pandemic [44]. It was not possible to conclusively assess whether these changes were also partly due to systematic deviations in the sociodemographic composition of the sample at the time of the survey [43]. It is also not possible to determine whether any seasonal fluctuations in depressive symptoms had an influence on the results because the survey periods were not identical to those of the comparison years. Nevertheless, the NAKO study provides particularly valuable evidence that an increase in depressive symptoms at the beginning of the pandemic was associated with social consequences of the pandemic, such as job loss, reduction of working hours without short-time allowance, but also overtime, as well as an increase in job insecurity and a worsening of the financial situation [43].

Our analyses, which differentiated by education (Table 4), indicate that in the first year of the pandemic, people in the low or medium education group in particular may have benefited at least temporarily, from pandemic-induced changes with regard to certain depression symptoms, as indicated by the reduction found in a stress-associated depression symptom in these groups. Nevertheless, both women and men in the low education group continued to be by far the most affected by this depression symptom during the pandemic. The education-specific decrease found in the frequency of the symptoms during the pandemic requires further research and explanation.

Other population-based studies of the pandemic have found a reduction in education-related differences in loneliness and life satisfaction [11, 12]. People with high education reported increased stress during the pandemic, so that existing educational differences in loneliness experiences and life satisfaction were reduced compared to the pre-pandemic period. Since the calculations of the cited studies apparently did not use age standardisation, the results must be interpreted with reservations in view of age-group-specific changes during the pandemic and different age structures of the education groups. Liu et al. [45] found, on the other hand, that people with few years of education had higher psychological stress at the beginning of the pandemic. However, it must be taken into account that no baseline level was determined in the study and that the data were not based on a random sample from the population, but on an online survey in which three times as many women as men participated, as well as a disproportionately high number of younger respondents. Nevertheless, the partly inconsistent findings confirm that there is a need for further research regarding this question of social inequalities in the consequences of the pandemic for mental health.

### 4.3 Strengths and limitations

The number of cases in GEDA 2019/2020 makes it possible to analyse the data on depressive symptoms and the associated individual symptoms by sex, age groups and education groups. In a departure from previous studies, any seasonal variations to be levelled out the two comparable time periods in 2019 and 2020. GEDA 2019/2020 is a survey with telephone interviews based on a random sample of landline and mobile phone numbers. Despite the weighting of the respective study population according to age, gender, region and education group according to the population composition at the corresponding time of the survey, deviations of the study population with regard to other characteristics cannot be ruled out [26].

The intended analysis by gender, age and education groups did not allow for a closer look at different phases during the pandemic.

Any fluctuations in the course of the pandemic with regard to depressive symptoms were highlighted in another publication [6].

The summary of the age group 15 to 29 years remains unsatisfactory in view of the considerable dynamics of life changes in this stage of life. Much higher case numbers would have been necessary for this. The same applies to certain life situations possibly negatively influenced by the COVID-19 pandemic, which could be associated with a higher risk of depressive symptoms, as could be shown, for example, in the special analysis of the NAKO health study [43].

How the discontinuation of support services (such as self-help groups) or, on the other hand, the creation of alternative support services (such as online formats for counselling and therapy), have affected mental health cannot be assessed on the basis of the GEDA data [10].

### 4.4 Conclusion

The adult population has proven to be predominantly resilient in terms of depressive symptoms during the period studied at the beginning of the pandemic. However, the results also indicate that there are large groups in the population that have to bear a higher symptom burden than others. This affects women to a greater extent than men and especially people with low and medium education. These results on the first year of the pandemic have shown that the frequency of depressive symptoms can be influenced by changes in the living environment and that high prevalences should not be accepted as circumstances that can be influenced little. It seems urgent to continue the data collection in order to further observe developments in the population and to determine whether and how the now cumulative crises, such as the pandemic, inflation and the consequences of the war in Europe since February 2022, affect mental health. Against the background of the current results, it is important to continue to observe how opportunities and risks develop for different age and education groups, women and men. Further analyses of the course in 2021 and 2022, which are already planned, will show whether the previous resilience has held up in the further course of the pandemic and the added crises, and whether the situation of the people aged 80 years and older has improved again. Supplementary surveys and analyses that also include children have been started, but will not be completed until mid-2023.

From 2022 onwards, any changes will no longer be interpreted in relation to the pandemic alone, because economic pandemic consequences and challenges, such as the war in Eastern Europe, could also have an impact on mental health.

## Key statements

- The prevalence of depressive symptoms decreased from 9.2% to 7.6% in the first period of the pandemic.
- The decrease in the prevalence of depressive symptoms can be attributed to individual symptom areas.
- The changes in the prevalence of individual symptoms differ between women and men as well as between age and education groups.
- The prevalence of individual symptoms is possibly influenced by changes in the living environment in times of the pandemic.
- The serious differences between education groups show the need, especially for women with low or medium education, to pay more attention in prevention to how they can be specifically supported and relieved.

## Figures and Tables

**Table 1:** Proportion of people with depressive symptoms according to PHQ-8 in the period before the COVID-19 pandemic, April 2019 to January 2020 (total N=10,220, women n=5,332, men n=4,863) compared to the period during the COVID-19 pandemic, April 2020 to January 2021 (total N=11,900, women n=6,338, men n=5,523)^*^ Source: GEDA 2019/2020

	April 2019 to January 2020			April 2020 to January 2021		
	n**	%	(95% CI)	n**	%	(95% CI)
**Total**	658	**9.2**	**(8.3–10.3)**	628	**7.6**	**(6.8–8.6)**
**Women (total)**	397	**9.8**	**(8.5–11.2)**	378	**7.6**	**(6.6–8.8)**
15–29 years	39	12.2	(8.5–17.2)	42	10.5	(7.3–14.8)
30–44 years	58	**10.4**	**(7.5–14.2)**	62	**6.6**	**(4.7–9.3)**
45–64 years	194	**11.3**	**(9.3–13.7)**	166	**8.2**	**(6.5–10.2)**
65–79 years	72	5.2	(3.8–7.0)	75	6.1	(4.3–8.4)
≥80 years	34	7.2	(4.1–12.3)	33	6.3	(3.8–10.1)
**Men (total)**	256	8.5	(7.2–10.0)	241	7.4	(6.1–8.8)
15–29 years	38	7.9	(5.3–11.8)	31	7.6	(4.9–11.5)
30–44 years	45	8.2	(5.8–11.4)	34	6.5	(4.1–10.1)
45–64 years	113	11.0	(8.5–14.0)	108	8.2	(6.3–10.7)
65–79 years	41	5.3	(3.4–8.4)	42	6.1	(3.6–10.2)
≥80 years	19	5.8	(3.3–10.0)	26	7.8	(4.4–13.6)

^*^ Due to missing values in the PHQ-8, 182 cases in 2019 and 268 in 2020 were not included in the analysis.

^**^ Number of persons with a positive PHQ-8 from the value range 10 and above

CI = confidence interval

Values in bold: p-value in t-test smaller than 0.05

**Table 2:** Age-standardised prevalence of depressive symptoms according to PHQ-8 in the period before the COVID-19 pandemic, April 2019 to January 2020 (total N=10,220, women n=5,332, men n=4,863) compared to the period during the COVID-19 pandemic, April 2020 to January 2021 (total N=11,900, women n=6,338, men n=5,523), by education^*^ Source: GEDA 2019/2020

	April 2019 to January 2020			April 2020 to January 2021		
	n**	%	(95% CI)	n**	%	(95% CI)
**Total**						
Low education group	178	13.4	(10.8–16.5)	152	10.2	(8.0–12.8)
Medium education group	337	**9.6**	**(8.3–11.0)**	318	**7.6**	**(6.5–8.8)**
High education group	141	3.9	(3.1–4.7)	158	3.8	(3.2–4.7)
**Women**						
Low education group	101	14.1	(10.3–19.2)	87	10.8	(7.8–14.9)
Medium education group	206	10.2	(8.4–12.2)	198	8.2	(6.8–9.9)
High education group	89	5.0	(3.8–6.5)	93	4.2	(3.3–5.4)
**Men**						
Low education group	75	11.6	(8.6–15.5)	63	9.0	(6.3–12.5)
Medium education group	129	8.9	(7.1–11.1)	113	6.7	(5.2–8.6)
High education group	51	2.7	(2.0–3.7)	65	3.5	(2.6–4.8)

^*^ Education group according to CASMIN classification

^**^ Number of persons with a positive PHQ-8 from the value range 10 and above

CI = confidence interval

Values in bold: p-value in t-test smaller than 0.05

**Table 3:** Proportion of people who were affected by the above symptoms (PHQ 1 to 8)* ‘on more than half the days’ or ‘nearly every day’ in the period before the COVID-19 pandemic, April 2019 to January 2020 (total N=10,220, women n=5,332, men n=4,863) compared to the period during the COVID-19 pandemic, April 2020 to January 2021 (total N=11,900, women n=6,338, men n=5,523) Source: GEDA 2019/2020

	**1. Little interest/lack of pleasure**				**2. Down/depressed/hopeless**			
	**April 2019 to January 2020**		**April 2020 to January 2021**		**April 2019 to January 2020**		**April 2020 to January 2021**	
	**%**	**(95% CI)**	**%**	**(95% CI)**	**%**	**(95% CI)**	**%**	**(95% CI)**
**Total**	9.7	(8.8–10.7)	9.8	(8.9–10.8)	7.3	(6.5–8.2)	6.6	(5.8–7.4)
**Women (total)**	9.0	(7.8–10.3)	10.0	(8.7–11.3)	7.3	(6.2–8.6)	6.6	(5.7–7.8)
15–29 years	8.2	(5.4–12.3)	11.5	(8.3–15.7)	8.8	(5.8–13.3)	9.2	(6.2–13.3)
30–44 years	8.9	(6.4–12.3)	8.7	(6.2–12.0)	**7.3**	**(5.0–10.5)**	**3.9**	**(2.5–6.1)**
45–64 years	9.9	(8.0–12.1)	10.4	(8.5–12.8)	7.7	(6.0–9.8)	7.2	(5.6–9.2)
65–79 years	8.2	(6.1–10.9)	8.0	(6.1–10.5)	5.1	(3.5–7.3)	6.5	(4.7–9.0)
≥80 years	8.8	(5.9–12.8)	12.4	(8.4–18.1)	7.9	(4.6–13.2)	6.9	(4.1–11.5)
**Men (total)**	10.5	(9.2–12.0)	9.3	(8.0–10.8)	7.2	(6.1–8.6)	6.3	(5.2–7.6)
15–29 years	9.0	(6.4–12.5)	9.2	(6.4–13.0)	5.1	(3.1–8.3)	7.0	(4.5–10.6)
30–44 years	11.6	(8.8–15.3)	7.4	(5.0–10.9)	7.8	(5.5–11.0)	5.1	(3.2–8.2)
45–64 years	12.6	(10.1–15.6)	10.3	(8.3–12.8)	8.9	(6.8–11.6)	7.6	(5.7–10.0)
65–79 years	6.6	(4.6–9.2)	6.9	(4.6–10.4)	5.1	(3.2–8.3)	4.1	(2.4–7.1)
≥80 years	9.1	(5.7–14.3)	16.0	(10.8–23.1)	7.4	(3.8–14.0)	6.6	(3.7–11.6)
	**3. Sleep disorders**				**4. Tired/loss of energy**			
	**April 2019 to January 2020**		**April 2020 to January 2021**		**April 2019 to January 2020**		**April 2020 to January 2021**	
	**%**	**(95% CI)**	**%**	**(95% CI)**	**%**	**(95% CI)**	**%**	**(95% CI)**
**Total**	21.2	(20.0–22.5)	20.0	(18.9–21.3)	**17.0**	**(15.9**–**18.2)**	**13.8**	**(12.7**–**14.8)**
**Women (total)**	23.4	(21.7–25.2)	23.4	(21.7–25.1)	**20.3**	**(18.6–22.1)**	**15.2**	**(13.8–16.7)**
15–29 years	21.0	(16.5–26.3)	18.4	(14.4–23.3)	**26.2**	**(21.2–31.9)**	**17.0**	**(13.2–21.7)**
30–44 years	18.7	(15.4–22.7)	18.3	(14.9–22.2)	**21.9**	**(18.1–26.1)**	**15.9**	**(12.8–19.6)**
45–64 years	25.7	(23.0–28.6)	24.4	(21.7–27.2)	**20.0**	**(17.5–22.8)**	**15.6**	**(13.4–18.1)**
65–79 years	24.6	(21.2–28.2)	24.6	(21.4–28.1)	**14.3**	**(11.6–17.5)**	**10.4**	**(8.4–12.8)**
≥80 years	28.8	(22.7–35.8)	37.4	(31.1–44.2)	18.5	(13.2–25.2)	18.3	(13.5–24.3)
**Men (total)**	19.0	(17.3–20.8)	16.6	(15.0–18.3)	13.6	(12.1–15.2)	12.0	(10.5–13.6)
15–29 years	**18.4**	**(14.5–23.1)**	**11.2**	**(8.3–14.9)**	10.9	(8.1–14.5)	12.0	(8.9–16.1)
30–44 years	14.9	(11.8–18.5)	11.4	(8.3–15.5)	14.6	(11.6–18.3)	11.5	(8.0–16.1)
45–64 years	21.3	(18.4–24.5)	20.2	(17.5–23.2)	16.0	(13.4–19.1)	11.4	(9.3–14.0)
65–79 years	18.6	(15.0–22.8)	20.5	(16.9–24.6)	10.9	(8.1–14.5)	11.6	(8.6–15.6)
≥80 years	24.3	(18.0–31.8)	22.3	(16.4–29.5)	11.3	(7.6–16.3)	16.9	(11.8–23.5)
	**5. Loss of appetite/overeating**				**6. Bad opinion, failure/disappointing family**			
	**April 2019 to January 2020**		**April 2020 to January 2021**		**April 2019 to January 2020**		**April 2020 to January 2021**	
	**%**	**(95% CI)**	**%**	**(95% CI)**	**%**	**(95% CI)**	**%**	**(95% CI)**
**Total**	**7.2**	**(6.4–8.1)**	**5.8**	**(5.1–6.6)**	**4.4**	**(3.8–5.2)**	**3.4**	**(2.8–4.1)**
**Women (total)**	7.3	(6.3–8.6)	6.2	(5.3–7.3)	**4.6**	**(3.6–5.7)**	**3.0**	**(2.4–3.8)**
15–29 years	**13.3**	**(9.8–17.9)**	**7.4**	**(5.0–10.8)**	9.2	(6.2–13.4)	6.3	(3.9–9.8)
30–44 years	7.5	(5.2–10.9)	5.0	(3.5–7.1)	7.0	(4.5–10.7)	4.2	(2.7–6.5)
45–64 years	6.9	(5.5–8.7)	6.6	(5.1–8.6)	3.4	(2.3–4.9)	2.2	(1.5–3.2)
65–79 years	3.6	(2.5–5.2)	5.3	(3.6–7.9)	1.3	(0.6–2.6)	1.4	(0.7–2.7)
≥80 years	4.2	(2.5–7.0)	7.6	(4.4–12.9)	0.6	(0.1–2.4)^1^	0.7	(0.3–1.9)^1^
**Men (total)**	**6.8**	**(5.7–8.2)**	**5.2**	**(4.2–6.4)**	4.2	(3.3–5.2)	3.7	(2.8–4.8)
15–29 years	9.3	(6.4–13.4)	7.3	(4.9–10.8)	7.5	(5.1–10.9)	6.6	(4.2–10.2)
30–44 years	7.3	(5.0–10.6)	5.9	(3.6–9.7)	5.5	(3.6–8.2)	4.3	(2.3–7.6)
45–64 years	6.9	(5.1–9.4)	4.8	(3.5–6.6)	2.8	(1.8–4.1)	3.2	(2.1–4.8)
65–79 years	4.1	(2.4–6.8)	3.1	(1.6–5.8)	1.5	(0.7–3.4)	1.2	(0.3–5.0)
≥80 years	4.1	(2.2–7.5)	2.9	(1.5–5.3)	3.6	(1.5–8.4)^1^	0.8	(0.2–2.9^1^)
	**7. Diminished ability to concentrate**				**8. Changes in movement or speech**			
	**April 2019 to January 2020**		**April 2020 to January 2021**		**April 2019 to January 2020**		**April 2020 to January 2021**	
	**%**	**(95% CI)**	**%**	**(95% CI)**	**%**	**(95% CI)**	**%**	**(95% CI)**
**Total**	5.1	(4.4–5.9)	4.9	(4.2–5.7)	3.7	(3.1–4.4)	3.4	(2.8–4.0)
**Women (total)**	5.2	(4.2–6.3)	4.0	(3.3–4.9)	3.3	(2.6–4.1)	2.9	(2.3–3.7)
15–29 years	7.8	(5.0–12.0)	5.4	(3.2–9.1)	3.4	(1.7–6.4)	2.1	(1.1–4.0)
30–44 years	4.7	(3.0–7.5)	3.1	(1.9–4.9)	3.6	(2.1–6.2)	3.5	(2.2–5.5)
45–64 years	5.8	(4.4–7.7)	4.9	(3.6–6.7)	3.8	(2.8–5.1)	4.2	(2.9–6.1)
65–79 years	1.8	(1.1–3.2)	1.9	(1.2–3.0)	2.7	(1.6–4.6)	1.7	(1.1–2.8)
≥80 years	5.7	(2.8–11.4)	4.7	(2.7–8.3)	1.4	(0.7–3.0)	1.0	(0.5–2.3)
**Men (total)**	4.9	(3.9–6.1)	5.7	(4.6–7.1)	4.1	(3.3–5.2)	3.7	(2.8–4.7)
15–29 years	6.1	(3.7–9.7)	6.8	(4.3–10.7)	4.9	(2.9–8.2)	3.7	(2.0–6.8)
30–44 years	4.4	(2.7–7.1)	5.8	(3.5–9.4)	3.2	(1.9–5.3)	3.1	(1.6–5.7)
45–64 years	5.7	(4.0–8.0)	5.2	(3.6–7.3)	5.1	(3.5–7.3)	4.1	(2.9–5.9)
65–79 years	3.1	(1.9–5.2)	3.6	(1.9–6.7)	2.8	(1.5–5.0)	3.8	(2.0–6.8)
≥80 years	**3.0**	**(1.3–6.6)**	**9.8**	**(5.5–17.0)**	3.3	(1.4–8.0^1^)	2.9	(1.2–6.9)

^*^ The full wording of the questioned symptoms can be found in section 2.2 depressive symptoms

^1^ Number of cases n<10

CI = confidence interval

Values in bold: p-value in t-test smaller than 0.05

**Table 4:** Age-standardised proportion of people who were affected ‘on more than half of the days’ or ‘almost every day’ by the symptoms mentioned (PHQ 1 to 8)* in the period before the COVID-19 pandemic, April 2019 to January 2020 (total N=10,220, women n=5,332, men n=4,863) compared to the period during the COVID-19 pandemic, April 2020 to January 2021 (total N=11,900, women n=6,338, men n=5,523), by education^**^ Source: GEDA 2019/2020

	**1. Little interest/lack of pleasure**				**2. Down/depressed/hopeless**			
	**April 2019 to January 2020**		**April 2020 to January 2021**		**April 2019 to January 2020**		**April 2020 to January 2021**	
	**%**	**(95% CI)**	**%**	**(95% CI)**	**%**	**(95% CI)**	**%**	**(95% CI)**
**Total**								
Low education group	13.8	(11.3–16.7)	13.6	(11.2–16.4)	10.2	(7.9–13.2)	8.9	(7.0–11.2)
Medium education group	9.7	(8.4–11.1)	9.0	(7.9–10.3)	7.8	(6.6–9.1)	6.3	(5.3–7.4)
High education group	5.1	(4.2–6.2)	5.4	(4.5–6.4)	3.5	(2.7–4.5)	3.5	(2.9–4.3)
**Women**								
Low education group	13.4	(9.9–18.0)	15.2	(11.6–19.7)	11.1	(7.5–16.2)	9.5	(6.9–13.1)
Medium education group	8.8	(7.2–10.7)	9.0	(7.6–10.8)	7.5	(6.0–9.4)	6.3	(5.1–7.7)
High education group	5.1	(3.9–6.5)	5.5	(4.3–6.9)	4.1	(3.1–5.6)	3.9	(3.0–5.1)
**Men**								
Low education group	13.8	(10.6–17.6)	10.9	(8.1–14.5)	8.8	(6.3–12.2)	7.7	(5.4–11.0)
Medium education group	10.6	(8.7–12.8)	8.8	(7.2–10.9)	8.0	(6.3–10.2)	6.2	(4.7–8.1)
High education group	5.2	(3.9–6.8)	5.3	(4.1–6.9)	2.8	(1.8–4.2)	3.1	(2.3–4.3)
	**3. Sleep disorders**				**4. Tired/loss of energy**			
	**April 2019 to January 2020**		**April 2020 to January 2021**		**April 2019 to January 2020**		**April 2020 to January 2021**	
	**%**	**(95% CI)**	**%**	**(95% CI)**	**%**	**(95% CI)**	**%**	**(95% CI)**
**Total**								
Low education group	25.1	(22.1–28.5)	23.4	(20.4–26.6)	**21.3**	**(18.3–24.6)**	**16.6**	**(13.9–19.7)**
Medium education group	21.0	(19.3–22.7)	19.1	(17.6–20.8)	**17.0**	**(15.5–18.7)**	**13.8**	**(12.4–15.3)**
High education group	14.2	(12.7–15.7)	13.8	(12.5–15.2)	10.5	(9.2–11.9)	9.2	(8.0–10.5)
**Women**								
Low education group	26.5	(21.9–31.7)	27.0	(22.8–31.7)	**25.5**	**(20.7–31.0)**	**18.3**	**(14.4–23.0)**
Medium education group	23.3	(21.0–25.8)	22.1	(20.0–24.4)	**20.4**	**(18.1–22.9)**	**15.9**	**(14.1–18.0)**
High education group	17.2	(15.0–19.6)	16.0	(14.1–18.2)	13.3	(11.2–15.6)	11.5	(9.6–13.7)
**Men**								
Low education group	23.4	(19.6–27.7)	19.7	(15.8–24.3)	16.6	(13.4–20.4)	14.1	(10.8–18.4)
Medium education group	18.7	(16.4–21.3)	16.0	(13.9–18.5)	13.7	(11.7–16.0)	11.6	(9.6–13.9)
High education group	11.1	(9.3–13.3)	11.5	(9.9–13.3)	7.6	(6.1–9.5)	6.7	(5.5–8.2)
	**5. Loss of appetite/overeating**				**6. Bad opinion, failure/disappointing family**			
	**April 2019 to January 2020**		**April 2020 to January 2021**		**April 2019 to January 2020**		**April 2020 to January 2021**	
	**%**	**(95% CI)**	**%**	**(95% CI)**	**%**	**(95% CI)**	**%**	**(95% CI)**
**Total**								
Low education group	10.3	(8.0–13.2)	7.5	(5.7–9.8)	7.5	(5.4–10.5)	4.6	(3.2–6.7)
Medium education group	7.6	(6.5–8.9)	6.1	(5.2–7.2)	4.9	(4.0–6.0)	3.7	(2.9–4.7)
High education group	3.9	(3.0–5.0)	2.6	(2.1–3.2)	2.1	(1.5–3.1)	2.0	(1.4–2.9)
**Women**								
Low education group	10.5	(7.2–15.0)	9.5	(6.7–13.4)	9.7	(6.0–15.2)	4.9	(2.9–8.3)
Medium education group	7.5	(6.0–9.2)	6.4	(5.2–7.8)	4.7	(3.5–6.2)	3.6	(2.6–4.9)
High education group	5.5	(4.0–7.6)	3.2	(2.4–4.1)	2.1	(1.3–3.6)	2.5	(1.5–4.1)
**Men**								
Low education group	9.1	(6.4–12.8)	5.6	(3.6–8.6)	4.9	(3.1–7.7)	4.3	(2.5–7.2)
Medium education group	7.7	(6.1–9.7)	5.8	(4.4–7.6)	5.0	(3.8–6.6)	3.7	(2.5–5.3)
High education group	2.2	(1.5–3.2)	2.0	(1.4–2.9)	2.1	(1.2–3.5)	1.6	(1.0–2.6)
	**7. Diminished ability to concentrate**				**8. Changes in movement or speech**			
	**April 2019 to January 2020**		**April 2020 to January 2021**		**April 2019 to January 2020**		**April 2020 to January 2021**	
	**%**	**(95% CI)**	**%**	**(95% CI)**	**%**	**(95% CI)**	**%**	**(95% CI)**
**Total**								
Low education group	7.2	(5.3–9.5)	6.2	(4.6–8.5)	5.1	(3.6–7.1)	5.2	(3.6–7.3)
Medium education group	5.1	(4.2–6.2)	4.9	(4.0–6.0)	3.7	(2.9–4.6)	3.3	(2.6–4.1)
High education group	2.0	(1.5–2.7)	2.5	(2.0–3.2)	1.6	(1.2–2.3)	1.8	(1.3–2.3)
**Women**								
Low education group	7.4	(4.9–11.2)	4.5	(2.7–7.4)	3.7	(2.0–6.8)	5.0	(2.9–8.4)
Medium education group	5.2	(4.0–6.9)	4.6	(3.5–6.0)	3.5	(2.6–4.8)	2.9	(2.1–4.1)
High education group	2.6	(1.7–4.0)	2.3	(1.6–3.2)	2.1	(1.4–3.3)	2.1	(1.5–3.1)
**Men**								
Low education group	6.5	(4.3–9.6)	7.5	(5.1–11.0)	6.0	(4.0–8.9)	5.0	(3.1–7.9)
Medium education group	4.9	(3.7–6.5)	5.1	(3.7–7.0)	3.9	(2.8–5.4)	3.5	(2.5–4.8)
High education group	1.4	(0.9–2.1)	2.7	(1.9–3.9)	1.1	(0.7–1.9)	1.3	(0.9–2.0)

^*^ The full text of the symptoms questioned in the PHQ-8 can be found in section 2.2 depressive symptoms

^**^ Education groups according to CASMIN classification

CI = confidence interval

Values in bold: p-value in t-test smaller than 0.05
